# My 100th JBC paper

**DOI:** 10.1016/j.jbc.2020.100061

**Published:** 2020-11-18

**Authors:** Aziz Sancar

**Affiliations:** Department of Biochemistry and Biophysics, University of North Carolina at Chapel Hill, Chapel Hill, North Carolina, USA

## Abstract

Nobel laureate Aziz Sancar writes about his decades-long relationship with the *Journal of Biological Chemistry*. Since 1984, he has published 100 papers in JBC, including this “Reflections.”

I just published my 99th *Journal of Biological Chemistry* (JBC) paper (1) and decided to reflect on my experience with the JBC as my 100th paper without waiting for the next research results to reach the 100th, which has been my holy grail since I became an independent researcher at the University of North Carolina at Chapel Hill in 1982.

During my graduate student education under the direction of Dr Claud S. Rupert at the University of Texas at Dallas, I mostly conducted photobiological work, and I was actually the first worker to introduce molecular biology/biological chemistry to the laboratory by cloning the photolyase gene (*phr*) and partially purifying the enzyme from cells carrying the cloned gene. Then, I went to the Yale University Department of Molecular Biophysics and Biochemistry to work in the laboratory of W. Dean Rupp on nucleotide excision repair in *Escherichia coli*. Dr Rupp, like Dr Rupert, had been trained as a DNA repair photobiologist, but in addition, he was using bacterial genetics and had recently become interested in cloning of DNA repair and recombination genes. To my knowledge, Dr Rupp, like Dr Rupert, had never purified a protein. Within 3 years in Dr Rupp’s laboratory, I cloned and purified *uvrA*, *B*, and *C* genes and proteins and the *ssb* and *recA* genes and proteins and published several papers on these subjects. Importantly, perhaps because neither my doctoral mentor nor my postdoctoral mentor was a biochemist, these and other early papers were not published in the JBC.

So, when my wife Gwendolyn and I joined the University of North Carolina Biochemistry Department in 1982, we were all of a sudden in a department in which most faculty members published their work in the JBC. I was, even as a graduate student, well acquainted with JBC, reading with a religious zeal Arthur Kornberg’s “Enzymatic Synthesis of Deoxyribonucleic Acids (I through XXXVI) (it was regarded by my generation as “The Bible”). So, when Gwen and I sequenced and purified photolyase, we sent our two papers for back-to-back publication in the JBC (received December 7, 1983, and published May 10, 1984) (2, 3). As a young investigator, with the usual anxieties associated with being a junior faculty member, this was a great morale boost. I felt that I had found a home for publishing my work in a high-quality journal with an editorial board of very distinguished biochemists.

After those back-to-back papers, I never looked back. My first choice has always been the JBC, though every now and then because of pressure from some of my students, postdocs, or collaborators, we have published in other journals as well. My experience with the JBC has been extremely satisfying. One particular case sticks in my mind: After 3 years of dedicated work, we sent three papers to the JBC to be considered for publication back to back. After a considerable delay (the papers were very long and covered areas ranging from enzyme purification to physical chemistry of DNA–protein interaction to photophysics of flavin-mediated catalysis), the reviews came back and, to our relief, were very positive. The papers were published on January 5, 1987, 6 months after submission (4, 5, 6). Years later, I ran into an eminent enzymologist who had expressed interest in photolyase and whom I had considered “competition” and therefore had excluded him as a potential reviewer of those three manuscripts. He complimented me on that work and asked me if we had received a review that simply said “This is going to be a benchmark in the field; publish without changing a word.” When I answered “yes,” he told me that he was that reviewer. Apparently, the associate editor was curious to know why this unknown upstart from the University of North Carolina was considering such a great scientist a competitor to be excluded from reviewing our submission.

I have many stories about our other JBC papers, though not all of them so dramatic. Still, I get nervous when the reviews are delayed by a few days and get excited when the reviews are constructive and positive. My students and postdocs find this behavior amusing, but, to me, the day you get a paper accepted by the JBC is a day for celebration (Fig. 1).

**Figure 1:**
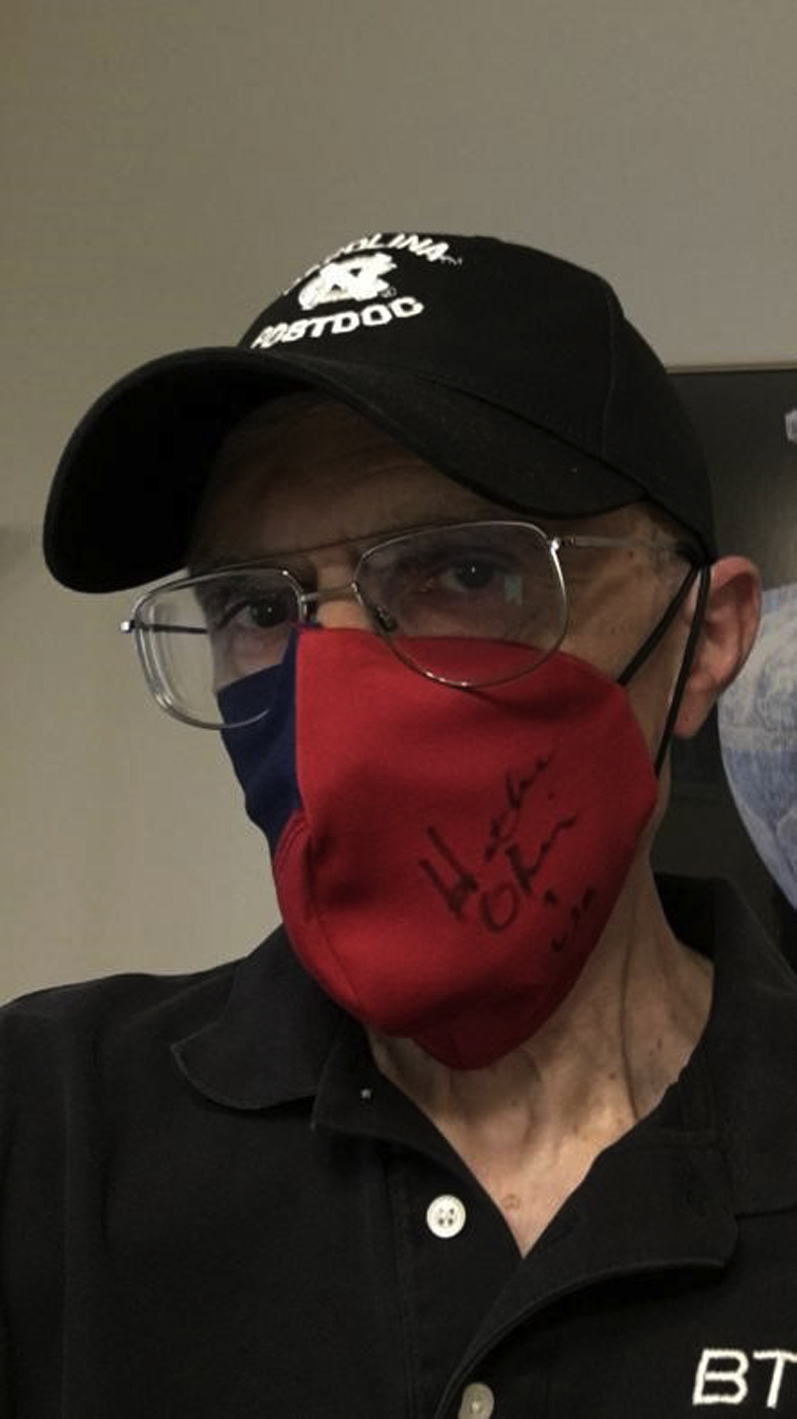
**During the COVID-19 pandemic, Aziz Sancar wears a mask made by three-time Olympic gold medalist and World Cup winner Heather O’Reilly.** The midfielder for the U.S. women’s soccer team made the mask for him from one of her jerseys. O’Reilly previously played at the University of North Carolina at Chapel Hill and led the Tar Heels to two National Collegiate Athletic Association championships and three Atlantic Coast Conference championships. Today, she’s an assistant coach at the University of North Carolina.

## Conflict of interest

The author declares no conflict of interest with the contents of this article.
